# Probiotic *Akkermansia muciniphila* alleviates acute kidney injury by protecting the intestinal barrier and modulating gut microbiota and metabolites

**DOI:** 10.7555/JBR.39.20250162

**Published:** 2025-05-28

**Authors:** Juan Ni, Zhan Yang, Xuewei Sun, Qian Cui, Ruonan Zhang, Han Lu, Zihan Wu, Jingfeng Zhu, Huijuan Mao, Kang Liu, Chengliang Tang, Chunhui Wang, Changying Xing, Jin Zhu

**Affiliations:** 1 Department of Nephrology, the First Affiliated Hospital of Nanjing Medical University, Jiangsu Province Hospital, Nanjing, Jiangsu 210029, China; 2 Department of Infectious Disease Prevention and Control, Huadong Medical Institute of Biotechniques, Nanjing, Jiangsu 210002, China; 3 Air Force Hospital of Eastern Theater, Nanjing, Jiangsu 210002, China; 4 School of Basic Medical Sciences, Nanjing Medical University, Nanjing, Jiangsu 211166, China; 5 Department of General Surgery, The Second Affiliated Hospital of Nanjing Medical University, Nanjing, Jiangsu 210011, China

**Keywords:** *Akkermansia muciniphila*, acute kidney injury, gut-kidney axis, metabolomics, gut microbiota dysbiosis

## Abstract

Acute kidney injury (AKI) is a critical condition with limited effective therapies. *Akkermansia muciniphila* (*A. muciniphila*) is a probiotic with multiple beneficial effects, including the regulation of epithelial cell tight junctions. Since renal pathophysiology is associated with gut barrier integrity, we hypothesized that *A. muciniphila* may have preventive effects on AKI. We established a lipopolysaccharide (LPS)-induced AKI mouse model to evaluate the effects of *A. muciniphila*. Our findings showed that pretreatment with *A. muciniphila* significantly attenuated kidney injury, as evidenced by reduced serum creatinine and urea nitrogen levels, alongside decreased tubular necrosis and apoptosis. *A. muciniphila* preserved intestinal barrier integrity and induced marked shifts in gut microbial ecology and the metabolome. *A. muciniphila* notably induced an increase in the relative abundance of the phylum *Proteobacteria* while decreasing in that of the phylum *Bacteroidetes*. At the genus level, *Prevotella*, *Faecalibaculum*, *Moraxella*, and *Lactobacillus* were more abundant in *A. muciniphila*-pretreated mice. Metabolomic analysis revealed that *A. muciniphila* altered the gut metabolome, with changes involving pathways such as tyrosine metabolism, alanine/aspartate/glutamate homeostasis, cancer-related carbon flux, and GABAergic synaptic signaling. In conclusion, our findings indicate that *A. muciniphila* exerts renoprotective effects by modulating the gut-kidney axis, thereby establishing a foundation for future studies to explore the connection between gut microbiota and AKI.

## Introduction

Acute kidney injury (AKI) is a prevalent and serious clinical condition characterized by a sudden loss of kidney function, affecting approximately 10%–15% of hospitalized patients and 50%–60% of those admitted to intensive care units^[1]^. Beyond its acute morbidity, AKI accelerates the progression to chronic kidney disease (CKD), with survivors facing a 2.3-fold increased risk of end-stage renal disease and mortality rates within 12 months surpassing 25%. One study analyzed AKI events across all stages in 3033 outpatients with CKD stages 3–5 enrolled in the CKD-REIN cohort study (2013–2020). Over a 3-year follow-up period, 443 patients experienced at least one AKI episode. Among these events, 27% were classified as stage 2 or 3 AKI, and 11% required dialysis. Furthermore, 74% of AKI cases involved hospitalization, with 47% of those being acquired during inpatient stays^[2]^. Given the lack of direct pharmacologic therapies for AKI, new therapeutic modalities are needed^[3–4]^.

With the development of biotechnology in microbial genomics, extensive research has linked gut microbiota to diseases such as liver disease^[5]^, obesity^[6]^, and diabetes mellitus^[7]^, as well as to the efficacy of cancer immunotherapy^[8]^. Renal disease is also correlated with the gut microbiota and its metabolites. Dysbiosis in CKD patients exacerbates CKD progression through gut-derived uremic toxin accumulation, which increases gut permeability, promotes endotoxemia and systemic inflammation^[9]^. Although studies on the interaction between AKI and gut microbial ecology have been limited, they are increasingly drawing research attention. AKI can induce microbial dysregulation and a leaky gut, and the gut microbiota can influence the severity of AKI^[10–11]^. These findings suggest that manipulating the gut microbiota and gut barrier function could serve as a potential therapeutic strategy for AKI. Current approaches to manipulate gut microbiota include exercise, prebiotics, probiotics, bacterial metabolites, fecal microbiota transplantation, bacteriophage therapy, and CRISPR–Cas9 editing^[9]^. Among these methods, the use of probiotics such as *Lactobacillus* and *Bifidobacterium* has the longest history, and new candidates have also emerged. For example, *Lactobacillus casei* Zhang alleviates both AKI and CKD in mice^[12]^. *Akkermansia muciniphila* (*A. muciniphila*), which was first isolated in 2004, shows unique potential with multiple beneficial effects. As a probiotic, *A. muciniphila* can reduce fat storage and insulin resistance and improve gut barrier function^[13]^. It can also protect against liver disorders^[14]^ and alleviate acute colitis. *A. muciniphila* has multiple mechanisms by which it improves gut barrier function, including the restoration of the mucus layer and an increase in the number of mucus-producing cells, as well as the regulation of epithelial cell tight junctions^[13,15]^. Given the correlation between AKI and gut barrier function, we hypothesized that *A. muciniphila* could ameliorate AKI by stabilizing the gut-kidney axis.

In this study, we established an AKI animal model by administering lipopolysaccharide (LPS) *via* intraperitoneal injection to C57BL/6 mice. We used the levels of creatinine and urea nitrogen in the peripheral blood of mice as standard indicators to assess renal function, complemented by interleukin (IL)-1, IL-6, and tumor necrosis factor-alpha (TNF-α) as markers of inflammation. *A. muciniphila* was administered to evaluate its effects on AKI.

## Materials and methods

### Bacterial strains

*A. muciniphila* (CICC24917) and *Bifidobacterium longum* (*B. longum*, CICC6181) were obtained from the China Center of Industrial Culture Collection (CICC, Beijing, China). *A. muciniphila* was cultured anaerobically in brain heart infusion (BHI) broth, while *B. longum* was cultured in de Man, Rogosa and Sharpe (MRS) broth (Becton, Dickinson and Company, Franklin Lakes, NJ, USA), both at 37 ℃ under strict anaerobic conditions. For *in vivo* administration, the *A. muciniphila* culture was harvested at mid-log phase, washed, and resuspended in sterile phosphate-buffered saline (PBS). The suspension was adjusted to a final concentration of 1 × 10^9^ colony-forming units (CFU) per 0.2 mL and administered to mice once daily *via* oral gavage.

### Experimental animals and treatment

To minimize variability associated with sex or estrogen levels, only C57BL/6 mice (4–6 weeks old) were used in this study. Mice were purchased from SiPeiFu Biotechnology Co., Ltd. (Beijing, China) and housed under specific-pathogen-free conditions with a 12-h light/dark cycle, constant temperature of 22 (± 2) ℃, and *ad libitum* access to standard rodent chow and water. Animals were acclimatized for seven days prior to experimentation, with six mice per cage. All procedures involving animals were approved by the Institutional Animal Care and Use Committee (IACUC) of the First Affiliated Hospital of Nanjing Medical University (IACUC-2103053).

**Figure 1:**
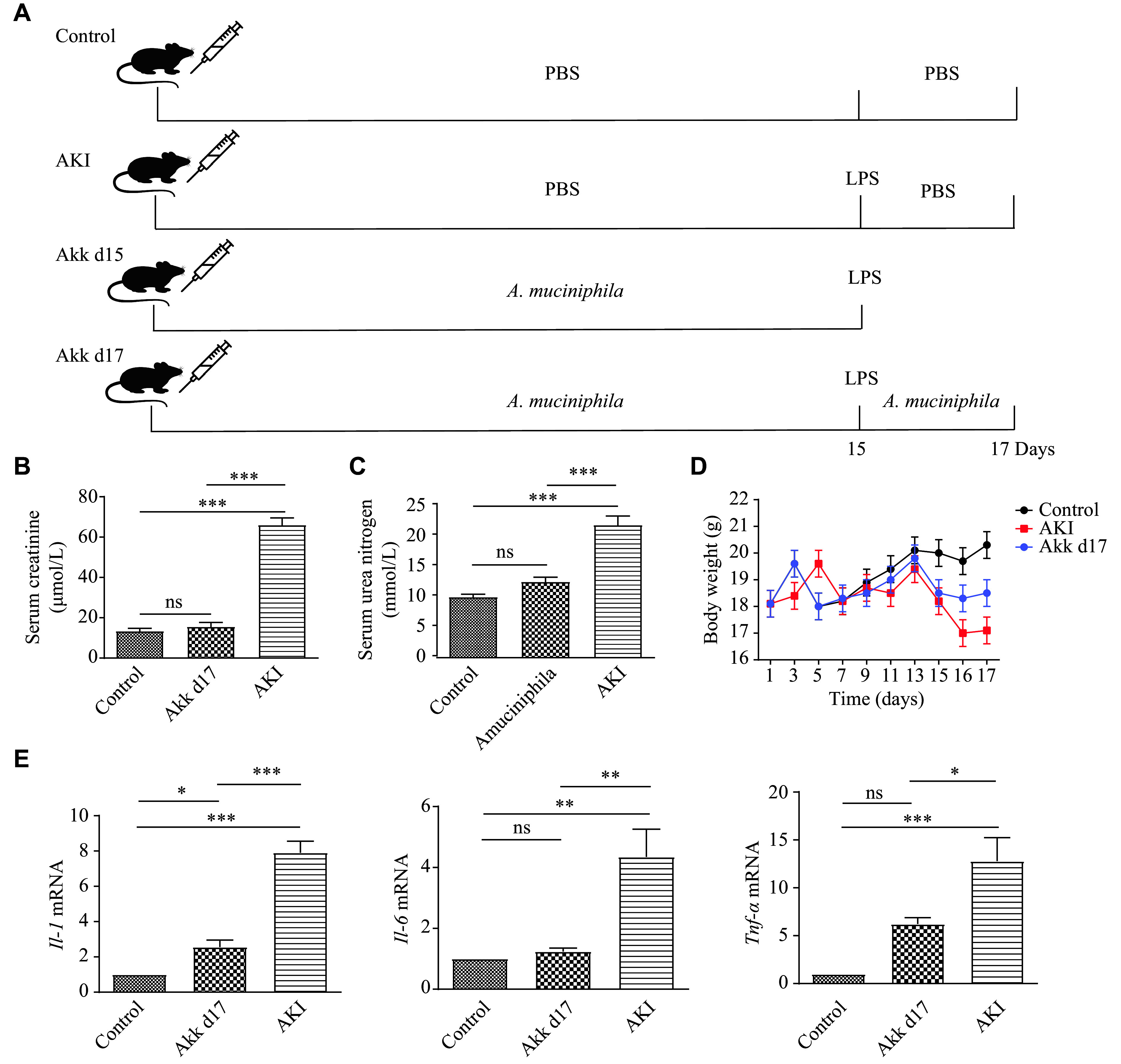
*A. muciniphila* alleviated LPS-induced AKI. A: Schematic overview of the experimental design. C57BL/6 mice in the Akk d15 and Akk d17 groups received daily oral gavage of *A. muciniphila* (1 × 10^9^ CFU) for 14 days, while mice in the control and AKI groups received daily gavage of sterile PBS (0.2 mL). On day 15, mice in the AKI, Akk d15, and Akk d17 groups received a single intraperitoneal injection of LPS (5 mg/kg body weight). Mice in the Akk d17 group continued to receive *A. muciniphila* for 2 days, while mice in the control and AKI groups continued to receive sterile gavage on days 16 and 17. B and C: Serum concentrations of creatinine (B) and urea nitrogen (C) were measured. D: Body weight of mice was monitored throughout the experiment. The weight of mice in the AKI group decreased rapidly, while the weight loss of the Akk d17 group was less than that of the AKI group. E: Relative mRNA expression levels of inflammatory cytokines (*Il-1*, *Il-6*, *Tnf-α*) in kidney tissues were quantified by real-time reverse transcription-PCR. Data are presented as mean ± standard deviation (*n* = 7 mice per group). ^*^P < 0.05, ^**^P < 0.01, and ^***^P < 0.001 by Student's *t*-test. Abbreviations: AKI, acute kidney injury; Akk, *A. muciniphila*; CFU, colony-forming units; IL-1, interleukin-1; IL-6, interleukin-6; LPS, lipopolysaccharide; TNF-α, tumor necrosis factor-alpha; ns, not significant.

Mice were randomly assigned to four groups (*n* = 7 per group): control, LPS-induced AKI, *A. muciniphila* d15 (Akk d15), and d17 (Akk d17) groups. Body weight was recorded every two days throughout the experiment. Mice in the Akk d15 and Akk d17 groups received daily oral gavage of *A. muciniphila* (1 × 10^9^ CFU) for 14 days, while mice in the control and AKI groups received daily gavage of sterile PBS (0.2 mL) for 14 days. On day 15, mice in the AKI, Akk d15, and Akk d17 groups received a single intraperitoneal injection of LPS (5 mg/kg body weight; Cat. #L2880, Sigma-Aldrich, St. Louis, USA). Mice in the Akk d17 group continued to receive probiotic administration for two additional days, while mice in the control and AKI groups received continuous PBS gavage on days 16 and 17 (***Fig. 1A***). All mice in the control, AKI and Akk d17 groups were euthanized on day 17.

Throughout the study, the health and growth status of the mice were assessed daily based on activity- and appearance-related parameters. Activity-related indicators included posture (*e.g.*, horizontal lying, prone position, body tilt, or head tilt), gait abnormalities (*e.g.*, paralysis, hypotonia, spasms, ataxia, or limping), mental state (ranging from hyperactivity to lethargy), and appetite (classified as excellent, good, reduced, or absent). Appearance-related indicators included ocular signs such as ptosis, conjunctival cloudiness, and exophthalmos, as well as coat condition evaluated for glossiness, thickness, presence of alopecia, and piloerection.

Mice in the Akk d15 group were euthanized on day 15, and mice in the other three groups were euthanized on day 17 under terminal anesthesia induced by intravenous injection of sodium pentobarbital. Peripheral blood was collected *via* retro-orbital sinus puncture, followed by centrifugation at 1500 *g* for 15 min at 4 ℃ to obtain serum. Stool samples were also collected. Additionally, kidney and colon tissues were harvested immediately after euthanasia.

### Renal function

Mouse serum was collected and equilibrated at 25 ℃ for 30 min in accordance with the manufacturer's instructions. Serum concentrations of creatinine and urea nitrogen were determined using a Mouse Creatinine (Cr) Assay Kit and a Urea Assay Kit (Jiancheng Bioengineering Institute, Nanjing, Jiangsu, China), respectively.

### Real-time reverse transcription-PCR (RT-qPCR)

To verify the expression of target genes (*Il-1*, *Il-6*, *Tnf-α*, *Muc2*), total RNA was extracted from mouse renal and colon tissues using the Fastagen RNA extraction kit (Shanghai, China). A NanoDrop 2000 spectrophotometer (Thermo Fisher, Waltham, MA, USA) was used to measure the quality (260/280 ratio) and quantity of RNA. The relative expression of target genes was normalized to *GAPDH*, and fold changes between groups were calculated using the 2^−ΔΔCt^ method. Fecal genomic DNA was extracted from colonic contents using the Qiagen PowerSoil DNA Isolation Kit (Qiagen, Duesseldorf, Germany) according to the manufacturer's instructions. The relative abundance of *A. muciniphila* was calculated using the 2^−ΔΔCt^ method. RT-qPCR was performed using the ABI 7500 real-time PCR system (Applied Biosystems, USA).

### Histopathological examination

Colon and kidney tissues were fixed in 4% formaldehyde for 24 h, followed by dehydration, clearing, paraffin infiltration, and embedding. Tissue blocks were sectioned at a thickness of 4 μm, with two sections mounted per slide. The sections were stained with hematoxylin and eosin (HE) and periodic acid-Schiff (PAS) reagents according to standard protocols.

For HE staining, deparaffinization was performed by baking slides at 65 ℃ for 30 min, followed by immersion in xylene twice for 10 min. Sections were hydrated through a graded ethanol series (100%, 95%, 85%, and 75%, 5 min each) and rinsed in distilled water. Nuclei were stained with hematoxylin for 10–15 min, followed by brief differentiation in acid alcohol and washing. Cytoplasmic staining was performed with alcoholic eosin for approximately 3 min. Finally, sections were dehydrated, cleared in xylene, and mounted.

For PAS staining, after deparaffinization and hydration, sections were oxidized in 1% periodic acid for 10–15 min, rinsed, and then treated with Schiff's reagent for 10–30 min, followed by a thorough water wash. Nuclei were counterstained with hematoxylin for 1–2 min, differentiated in 1% acid alcohol, and blued in ammonia water. After a final wash, sections were dehydrated, cleared, and mounted for evaluation.

PAS staining highlights glomerular and tubular basement membranes and mesangial matrix. Pathological changes were evaluated under a light microscope by a pathologist in a blinded manner. For histopathological scoring based on HE-stained kidney sections, the percentage of tubules exhibiting tubular dilatation, epithelial cell necrosis, interstitial edema, and loss of brush border was assessed and scored semi-quantitatively as follows: 0, none; 1, 0%–20%; 2, > 20%–50%; 3, > 50%–70%; 4, > 70%.

### Immunohistochemistry analysis

Paraffin-embedded tissue sections were deparaffinized by immersion in xylene three times for 9 min each, followed by rehydration through a graded ethanol series (100% and 95%) and five rinses in PBS. Endogenous peroxidase activity was quenched by incubation in 3% hydrogen peroxide for 10 min, followed by five additional PBS washes. Antigen retrieval was performed using sodium citrate buffer (pH 6.0) in a microwave oven at 700 W for 15 min. Sections were then blocked and subsequently incubated overnight at 4 ℃ in a humidified chamber with the following primary antibodies: monoclonal anti-BAX (1∶200; Cat. #14796, Cell Signaling Technology, Danvers, MA, USA), monoclonal anti-BCL-2 (1∶250; Cat. #ab182858, Abcam, Cambridge, UK), and polyclonal anti-KIM-1 (1∶100; Cat. #NBP1-76701, Novus Biologicals, Littleton, CO, USA). After washing, immunoreactivity was detected using an HRP-conjugated polymer-based detection system. The chromogen 3,3*'*-diaminobenzidine (DAB; Cat. #K3468, Dako, Denmark) was used for color development, followed by hematoxylin counterstaining. Digital images of the renal cortex and medulla were acquired using an Eclipse Ni light microscope (Nikon Co., Ltd., Japan). The percentage of positively stained area for KIM-1, BAX, and BCL-2 within a defined field of view (×200 magnification) was quantified using NIS-Elements imaging software (DS-Ri2 camera, Nikon Co., Ltd., Japan) and expressed as the positive area per unit area.

### Western blotting

Total proteins were extracted from colonic tissue using a Total Protein Extraction Kit (Bestbio, Shanghai, China) and quantified using a bicinchoninic acid assay kit (Vazyme, Nanjing, China). Equal amounts of protein (at least 10 μg per lane) were separated by 10% SDS-PAGE and transferred onto nitrocellulose membranes. Following blocking with 5% skim milk at room temperature for 2 h, the membranes were incubated overnight at 4 ℃ with primary antibodies against MUC-2 (1∶1000; Abcam, Shanghai, China) and β-actin (1∶1000; Cell Signaling Technology, Danvers, MA, USA). After three washes with TBST, the membranes were probed with HRP-conjugated goat anti-rabbit IgG (H+L) secondary antibody (1∶2000; Cell Signaling Technology) at room temperature for 1 h. Immunoreactive bands were detected using a chemiluminescent substrate, and images were captured using a Tanon imaging system (Tanon, Shanghai, China). Band intensities were quantified by densitometric analysis using ImageJ software.

### Intestinal permeability assay

After a 4-hour fast, mice were orally gavaged with fluorescein isothiocyanate (FITC)-dextran (FD4, 4 kDa; 500 mg/kg body weight; Sigma-Aldrich, St. Louis, MO, USA) to assess intestinal mucosal barrier permeability. One hour post-gavage, approximately 100 μL of blood was collected *via* tail vein puncture by clipping 1 cm from the tail tip into serum collection tubes. Mice were then euthanized by intravenous injection of sodium pentobarbital. Blood samples were centrifuged at 10000 *g* for 10 min at room temperature to obtain serum, which was subsequently diluted 1∶4 with ultrapure water. A standard curve was generated using serial dilutions of FITC-dextran in water (1∶300, 1∶1000, 1∶3000, 1∶10000, 1∶30000, 1∶100000, 1∶300000, 1∶1000000, and 1∶3000000). Both diluted serum samples and standards (100 μL per well) were loaded into a 96-well plate. Fluorescence intensity was measured using a microplate reader (Tecan Spark, Tecan Group Ltd., Männedorf, Switzerland) with excitation at 485 nm and emission at 528 nm. Intestinal permeability was calculated from the standard curve and multiplied by four to correct for the initial serum dilution.

To assess bacterial translocation, mesenteric lymph nodes (approximately 10 mg per sample) were aseptically harvested from mice and homogenized in 1 mL of sterile PBS using a sterile tissue grinder. A 100-μL aliquot of the diluted homogenate was plated onto MacConkey agar and incubated aerobically at 37 ℃ for 24 h. Bacterial colony formation was subsequently examined to assess the presence and extent of bacterial translocation.

### 16S rRNA gene sequence

Fecal samples collected prior to euthanasia were used for 16S rRNA gene sequencing. Genomic DNA was extracted from colonic contents using the Qiagen PowerSoil DNA Isolation Kit (Qiagen, Germany) following the manufacturer's instructions. The V3–V4 hypervariable regions of the bacterial 16S rRNA gene were amplified using universal primers (343F: 5*'*-TACGGRAGGCAGCAG-3*'* and 798R: 5*'*-AGGGTATCTAATCCT-3*'*). Amplicons were purified with Agencourt AMPure XP magnetic beads (Beckman Coulter, Brea, CA, USA), subjected to a second indexing PCR, and purified again using the same bead-based method. Final libraries were quantified using a Qubit fluorometer and sequenced on the Illumina NovaSeq 6000 platform (OE Biotech Co., Ltd., Shanghai, China). Paired-end reads were quality-filtered using Trimmomatic, then merged, denoised, and clustered into operational taxonomic units (OTUs) using QIIME and VSEARCH. Taxonomic annotation of OTUs was performed using the SILVA reference database. Alpha diversity was assessed based on Shannon and Chao1 indices, while beta diversity was visualized *via* principal coordinate analysis (PCoA) and linear discriminant analysis effect size (LEfSe). All statistical analyses and visualizations were implemented in R (version 4.1.2).

### Metabolomic analysis by gas chromatography-mass spectrometry (GC-MS)

Fresh fecal samples were collected from mice into sterile containers and immediately stored at −80 ℃ until processing. For metabolite extraction, 60 mg of feces was accurately weighed into a 1.5 mL centrifuge tube containing 40 μL of internal standard solution. Pre-chilled methanol (360 μL) and two stainless-steel beads were added, and the tubes were incubated at −20 ℃ for 5 min. Samples were homogenized using a tissue grinder (60 Hz, 2 min), followed by ultrasonic extraction in an ice-water bath for 30 min. Subsequently, 200 μL of chloroform and 400 μL of ultrapure water were added, and the mixture was vortexed for 2 min. After a second round of ultrasonication (30 min, ice bath), samples were incubated at −20 ℃ for 30 min and then centrifuged at 16000 *g* at 4 ℃ for 10 min. A 150 μL aliquot of the upper aqueous phase was transferred to a glass vial and dried under vacuum using a centrifugal concentrator. Dried residues were reconstituted with 80 μL of methoxylamine hydrochloride in pyridine (15 mg/mL), vortexed for 2 min, and incubated at 37 ℃ with shaking for 90 min to allow oximation of carbonyl groups. Thereafter, 50 μL of N,O-bis(trimethylsilyl)trifluoroacetamide containing 1% trimethylchlorosilane, 20 μL of n-hexane, and 10 μL of additional internal standard were added. The mixture was vortexed for 2 min and derivatized at 70 ℃ for 60 min.

Derivatized samples were equilibrated at room temperature for 30 min prior to GC-MS analysis. Analyses were performed on an Agilent 7890B gas chromatograph coupled to an Agilent 5977B mass selective detector (Agilent Technologies Inc., Santa Clara, CA, USA). Separation was carried out on an HP-5MS fused-silica capillary column (30 m × 0.25 mm i.d., 0.25 μm film thickness; Agilent, Folsom, CA, USA) with helium (purity > 99.999%) as carrier gas at a constant flow rate of 1 mL/min. The injection volume was 1 μL in splitless mode, and the injector temperature was set to 260 ℃. The oven temperature program was as follows: initial hold at 60 ℃ for 0.5 min, ramp to 125 ℃ at 8 ℃/min, then to 210 ℃ at 5 ℃/min, to 270 ℃ at 10 ℃/min, and finally to 305 ℃ at 20 ℃/min, with a final hold at 305 ℃ for 5 min. The quadrupole and electron impact ion source temperatures were maintained at 150 ℃ and 230 ℃, respectively. Electron ionization was performed at 70 eV, and mass spectra were acquired in full-scan mode (m/z 50–500) with a solvent delay of 300 s.

Raw data were processed using Agilent MassHunter software, and the resulting metabolomics data matrix was imported into R (version 4.1.2) for multivariate statistical analysis. Principal component analysis (PCA) was performed to assess overall metabolic variation and analytical reproducibility. Group discrimination was further evaluated using partial least squares discriminant analysis (PLS-DA).

### Statistical analysis

Data are presented as mean ± standard deviation. Differences among multiple groups were evaluated by one-way analysis of variance, followed by the Newman–Keuls post hoc test for pairwise comparisons. Metabolomics data processing and multivariate statistical analyses were performed using MetaX software. Additional statistical analyses and graphical visualization were carried out using GraphPad Prism (version 5.0) and R (version 4.1.2). A two-tailed *P*-value < 0.05 was considered statistically significant.

## Results

### *A. muciniphila* alleviated AKI in mice

We established an LPS-induced AKI mouse model, as shown in ***Fig. 1A***. The concentrations of serum creatinine and urea nitrogen were compared among the groups. The mice in the AKI group had significantly higher levels of creatinine and urea nitrogen than those in the other two groups, whereas there were no significant differences between the Akk d17 and control groups (***Fig. 1B*** and ***1C***). Specifically, the average levels of creatinine and urea nitrogen in the AKI group were 66.11 (± 3.75) μmol/L and 21.28 (± 1.59) mmol/L, respectively, while the average levels of creatinine in the Akk d17 group and the control group were 15.60 (± 2.45) μmol/L and 13.49 (± 1.66) μmol/L, respectively, and the average levels of urea nitrogen in these two groups were 12.45 (± 2.38) mmol/L and 10.10 (± 1.52) mmol/L, respectively. The body weight of the mice in the AKI group (17.11 [± 0.49] g) was significantly lower than that of the control group (20.13 [± 0.61] g) and the Akk d17 group (18.31 [± 0.59] g) on day 17 (***Fig. 1D*** and ***Supplementary Fig. 1***). The mice in the AKI group also had a poor appetite, slow gait, hair loss, and cloudy conjunctiva (***Supplementary Fig. 2***). However, these symptoms were alleviated in the Akk d17 group. Quantification of *Il-1*, *Il-6*, and *Tnf-α* mRNA in the renal tissue showed that their expression levels were higher in the AKI group than in the other groups (***Fig. 1E***).

**Figure 2:**
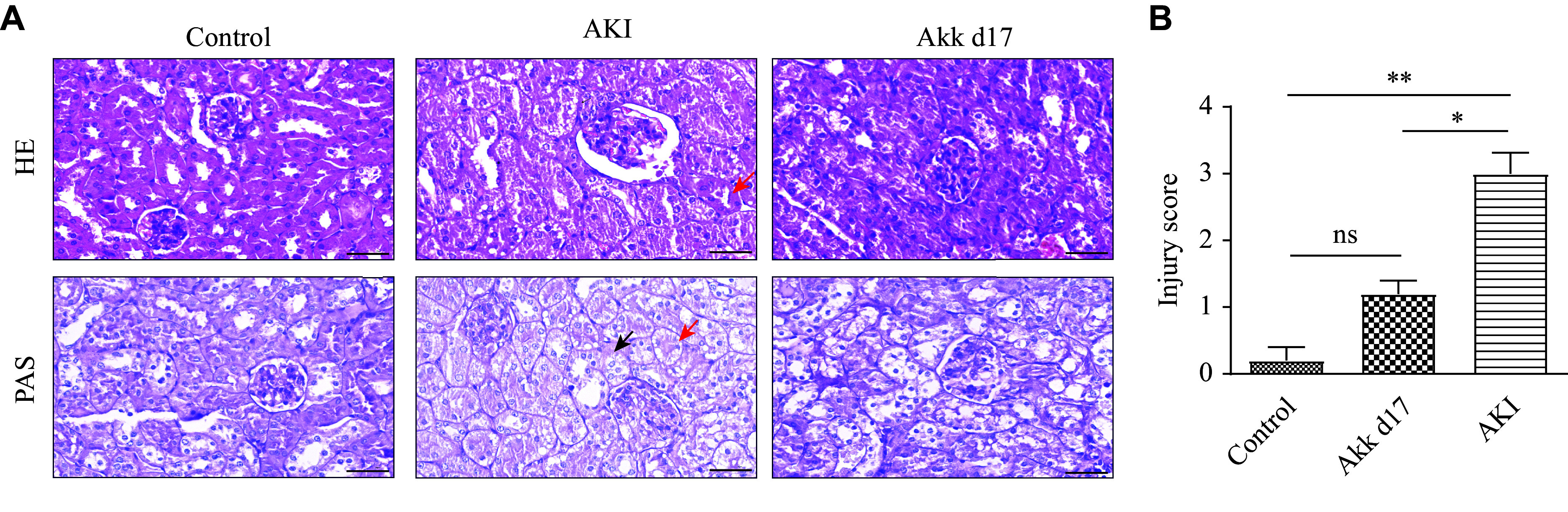
*A. muciniphila* reduced renal cell injury in mice with LPS-induced AKI. A: Kidney sections from each experimental group were stained with HE and PAS. Scale bar, 50 μm. It showed cellular necrosis (red arrows) and the loss of brush border (black arrows) in the AKI group. B: Injury scores were calculated based on the percentages of tubules showing tubule dilatation, cellular necrosis, interstitial edema, and loss of brush border as follows: 0, none; 1, 0%–20%; 2, 20%–50%; 3, 50%–70%; 4, > 70% (based on HE staining). Data are presented as mean ± standard deviation (*n* = 7 for each group). ^*^P < 0.05 and ^**^P < 0.01 by Student's *t*-test. Abbreviations: AKI, acute kidney injury; Akk, *A. muciniphila*; HE, hematoxylin and eosin; LPS, lipopolysaccharide; PAS, periodic acid–Schiff; ns, not significant.

The degree of renal injury was assessed by HE and PAS staining (***Fig. 2A***). Renal sections of mice in the AKI group exhibited increased apoptosis; granular tubular epithelial cells with vacuolar degeneration; loss of brush-border microvilli; dilation of tubular lumens; and interstitial edema. The injury to the renal tubular epithelium was alleviated in the Akk d17 group, indicating recovery. Based on the HE staining, we assigned an injury score. While the AKI group scored much higher than the Akk d17 group, the Akk d17 and control groups had comparable scores with no significant difference (***Fig. 2B***).

**Figure 3:**
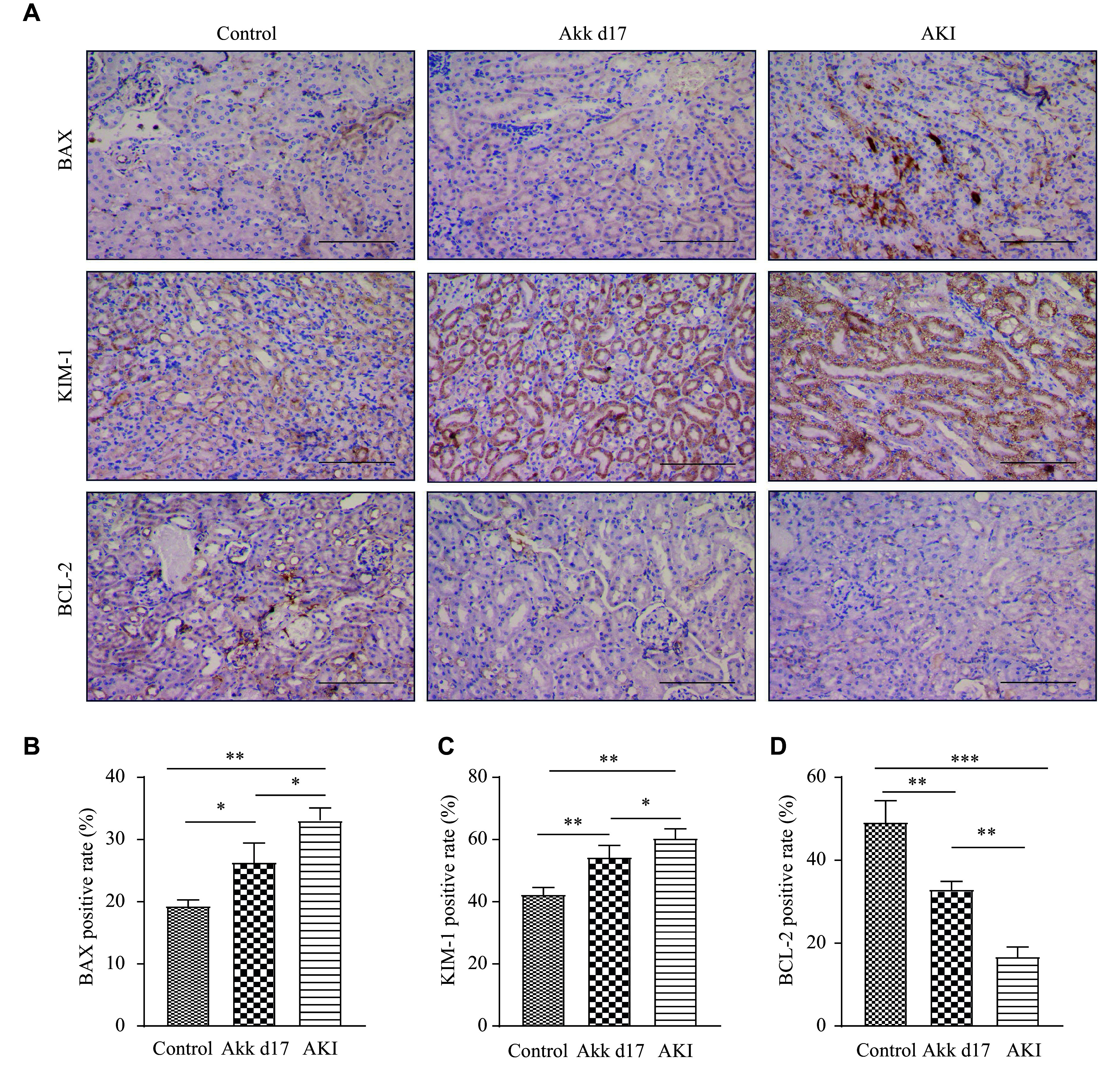
*A. muciniphila* protected against apoptosis in LPS-induced AKI. A: Mouse kidney tissues from the control, AKI, and Akk d17 groups were analyzed by immunohistochemical staining. Scale bar, 100 μm. B–D: Semi-quantitative analysis showing the expression of BAX (B), KIM-1 (C), and BCL-2 (D). Data are presented as mean ± standard deviation (*n* = 7 for each group). Statistical analyses were performed using Student's *t*-test. ^*^P < 0.05, ^**^P < 0.01, and ^***^P < 0.001. Abbreviations: AKI, acute kidney injury; Akk, *A. muciniphila*; BCL-2, B-cell lymphoma 2; BAX, BCL-2-associated X protein; KIM-1, kidney injury molecule 1; LPS, lipopolysaccharide.

The expression levels of BAX, KIM-1, and BCL-2 proteins were examined by IHC (***Fig. 3A***–***3D***). Specifically, the positive rates of BAX, KIM-1, and BCL-2 in the AKI group were 33%, 60%, and 17%, respectively; those in the Akk d17 group were 26%, 57%, and 31%, respectively; and those in the control group were 19%, 42%, and 49%, respectively. The AKI group showed higher BAX and KIM-1 expression levels than the Akk d17 group. BCL-2 expression was lower in the AKI group than in the Akk d17 group. These findings suggest that *A. muciniphila* reduces AKI-related tubule cell injury and may protect the cells from apoptosis.

To further illustrate the biological effects of *A. muciniphila*, we used heat-treated *A. muciniphila* and *B. longum* as controls. In contrast to *A. muciniphila*, heat-treated *A. muciniphila* and *B. longum* did not reduce the serum creatinine and urea nitrogen levels (***Supplementary Fig. 3***). Moreover, HE and PAS staining also showed kidney injury in the heat-treated *A. muciniphila* and *B. longum* groups (***Supplementary Fig. 4***). These results demonstrated that *A. muciniphila* alleviated LPS-induced AKI in mice.

### *A. muciniphila* altered the gut microbiota composition in AKI mice

**Figure 4:**
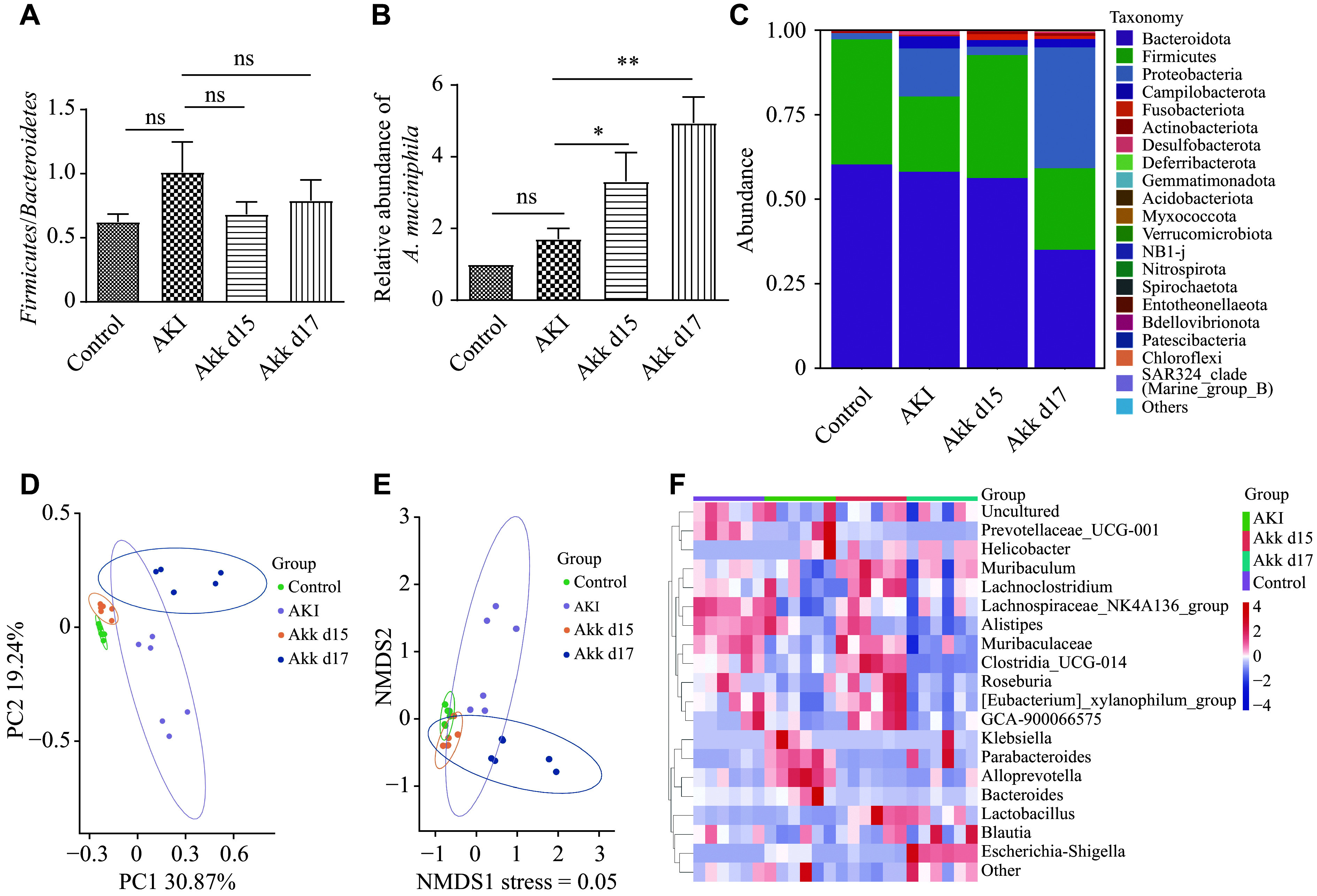
*A. muciniphila* altered the gut microbiota composition as it alleviated AKI. Fecal samples were collected for 16S rRNA gene sequencing. Genomic DNA was extracted from colonic contents. Alpha diversity was assessed based on Shannon and Chao1 indices, while beta diversity was visualized *via* principal coordinate analysis and linear discriminant analysis effect size. A: *Firmicutes*/*Bacteroidetes* ratios among the four groups. B: Relative abundance of *A. muciniphila* (based on 16S rRNA gene sequences) in the four groups. C: The gut microbiota at the phylum level in the four groups. D and E: Principal component analysis (PCA) and non-metric multidimensional scaling (NMDS) analysis of the gut microbiota. F: The heatmap shows the differences in microbiota composition across the four groups. Data are presented as mean ± standard deviation (*n* = 6 mice per group). Differences among multiple groups were evaluated by one-way analysis of variance, followed by the Newman–Keuls post hoc test for pairwise comparisons. ^*^P < 0.05 and ^**^P < 0.01. Abbreviations: AKI, acute kidney injury; Akk, *A. muciniphila*; ns, not significant.

We compared the gut microbiota among the control, AKI, Akk d15, and Akk d17 groups by 16S rRNA sequencing. The *Firmicutes*/*Bacteroidetes* (F/B) ratio was used to assess the relative abundance of two dominant bacterial phyla in the gut, which has been associated with metabolic status, the gut ecosystem, and numerous diseases. The F/B ratio of the AKI group (1.01) was higher than those of the Akk d15 (0.68), Akk d17 (0.79), and control group (0.62), although the difference was not significant (***Fig. 4A***). At the phylum level, the Akk d17 group had a higher proportion of *Proteobacteria* than the AKI group, while the Akk d17 and Akk d15 groups had a lower proportion of *Bacteroidota* than the AKI group (***Fig. 4C***). PCA and non-metric multidimensional scaling (NMDS) analyses showed that the overall gut microbiome composition differed among the four groups (***Fig. 4D*** and ***4E***), and the heatmap also demonstrated these differences (***Fig. 4F***). Moreover, we identified the bacteria that were significantly different between the Akk d17 and AKI groups (***Supplementary Fig. 5***). *Alloprevotella*, *Bacteroides*, *Anaerovorax*, *Alistipes*, and *Ideonella* were highly abundant in the AKI group, while *Prevotella*, *Fusobacterium*, *Faecalibaculum*, *Leptotrichia*, *Acinetobacter*, *Moraxella*, and *Lactobacillus* were highly abundant in the Akk d17 group. RT-qPCR analysis showed the relative abundance of *A. muciniphila* in the four groups and revealed a higher abundance in the Akk d17 and Akk d15 groups than in the other two groups (***Fig. 4B***).

### *A. muciniphila* altered the fecal metabolite composition in AKI mice

**Figure 5:**
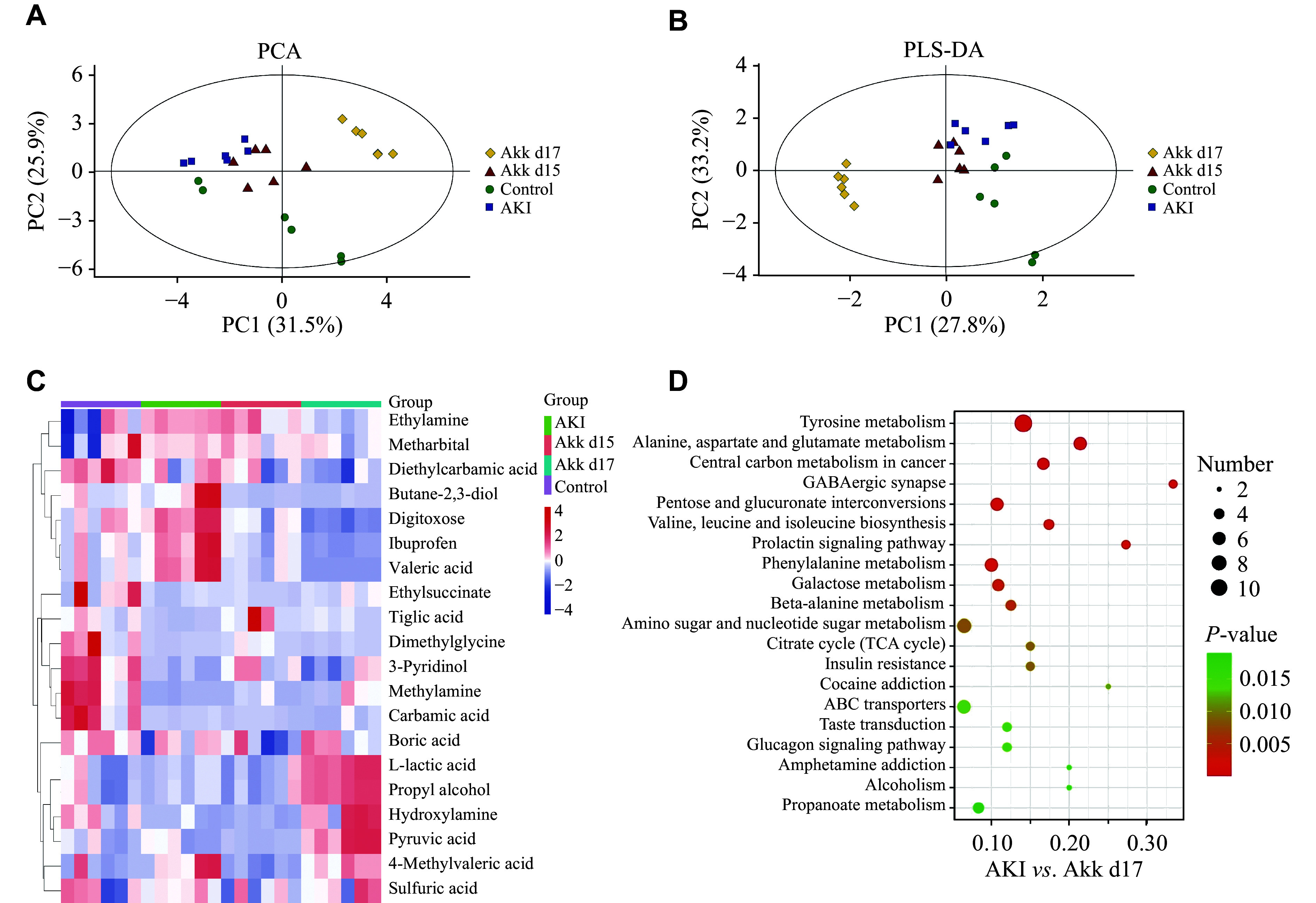
*A. muciniphila* altered the metabolome of the gut microbiota. Metabolic profiles were analyzed by GC-MS. Multivariate analyses were performed on fecal metabolomes from mice in the control, AKI, Akk d15, and Akk d17 groups. Score plots from principal component analysis (PCA; A) and partial least squares discriminant analysis (PLS-DA; B) show separation among the four groups. C: A heatmap depicting the relative abundance of differentially expressed metabolites across the four experimental groups. Data are presented as *Z*-scores based on GC-MS analysis of fecal samples. D: Altered metabolic pathways were identified by comparative analysis between the AKI group and the Akk d17 group. *n* = 6 mice per group. Abbreviations: AKI, acute kidney injury; Akk, *A. muciniphila*; GC-MS, gas chromatography-mass spectrometry; PCA, principal component analysis; PLS-DA, partial least squares-discriminant analysis.

We employed GC-MS to analyze the fecal metabolites in the control, AKI, Akk d15, and Akk d17 groups. PCA and OPLS-DA analyses showed differences in the metabolite content among the four groups (***Fig. 5A*** and ***5B***). The heatmap from the hierarchical clustering analysis displayed the differentially abundant metabolites in the four groups (***Fig. 5C***). The AKI group exhibited a higher abundance of butane-2,3-diol, digitoxose, ibuprofen, and valeric acid than the other three groups. In contrast, the Akk d17 group had elevated levels of L-lactic acid, propyl alcohol, hydroxylamine, pyruvic acid, 4-methyl valeric acid, and sulfuric acid compared with the other three groups. Based on the KEGG database, we observed that the differential metabolite levels between the AKI and Akk d17 groups were enriched in pathways related to tyrosine metabolism, alanine, aspartate, and glutamate metabolism, central carbon metabolism in cancer, GABAergic synapse, pentose and glucuronate interconversions, valine, leucine and isoleucine biosynthesis, prolactin signaling, phenylalanine metabolism, and galactose metabolism (***Fig. 5D***).

### *A. muciniphila* protected the gut barrier and alleviated intestinal injury in AKI mice

**Figure 6:**
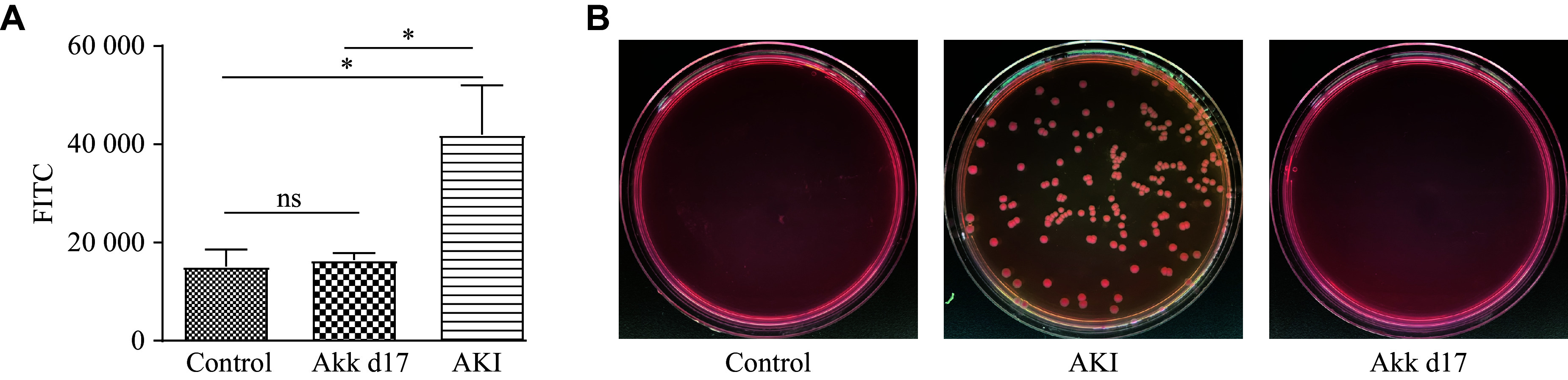
*A. muciniphila* promoted gut barrier functions. A: Fluorescence intensity was measured following oral gavage with FITC-dextran (500 mg/kg body weight), using an enzyme labeling instrument with excitation at 485 nm and emission at 528 nm. Intestinal permeability was calculated from the standard curve and multiplied by four to correct for the initial serum dilution. Data are presented as mean ± standard deviation (*n* = 7 for each group). Statistical analyses were performed using Student's *t*-test. B: Mesenteric lymph nodes (approximately 10 mg per sample) were aseptically harvested from mice in each group and homogenized in 1 mL of sterile PBS using a sterile tissue grinder. One hundred microliters of diluted homogenate was plated onto MacConkey agar and incubated aerobically at 37 ℃ for 24 h. Bacterial colony formation was subsequently examined to assess bacterial translocation. ^*^P < 0.05. Abbreviations: AKI, acute kidney injury; Akk, *A. muciniphila*; FITC, fluorescein isothiocyanate; ns, not significant.

After oral administration of FITC-dextran, we measured the fluorescence intensity of the serum. The AKI group had higher serum fluorescence than the control and Akk d17 groups (***Fig. 6A***), indicating that the gut barrier of mice in the AKI group was severely damaged compared with those in both the control and Akk d17 groups. We also assessed bacterial translocation through the gut barrier by culturing bacteria from the mesenteric lymph nodes. While cultures of bacteria from the AKI group yielded several colonies, those from the Akk d17 group yielded very few (***Fig. 6B***), indicating that *A. muciniphila* protected the gut barrier from LPS-induced damage. The AKI group had lower *Muc2* gene and protein expression than the other three groups, while the Akk d15 group showed the highest expression. Although the MUC2 expression in the Akk d17 group was lower than that in the Akk d15 group, it was still higher than that in the AKI group (***Supplementary Fig. 6***). *A. muciniphila* also alleviated the intestinal injury induced by LPS. The AKI group exhibited necrosis of colon crypt epithelial cells and the presence of serous material in the small intestinal glands, while the Akk d17 group exhibited less severe injury (***Supplementary Fig. 7***).

## Discussion

In the present study, we investigated interventions for AKI from the perspective of the gut and probiotics, an area that currently remains under-researched. Additionally, some studies have investigated novel drugs for AKI, such as those targeting α-amino-β-carboxymuconate-ε-semialdehyde decarboxylase. We demonstrated that *A. muciniphila* alleviated LPS-induced AKI in a mouse model. Specifically, this probiotic reduced the concentrations of serum creatinine and urea nitrogen, lowered the mRNA levels of *Il-1*, *Il-6*, and *Tnf-α* in renal tissues, and alleviated cell damage in AKI mice.

A previous study reported an alteration in the gut microbiome in ischemia/reperfusion-induced AKI. Most notably, there was an increase in *Enterobacteriaceae* and a decrease in *Lactobacilli* and *Ruminococacceae*^[10]^. In the present study, we found that *A. muciniphila* also changed the gut microbiome composition at the genus level, increasing the abundance of *Faecalibaculum*. *Faecalibacterium prausnitzii*, the primary producer of butyrate in the gut, has many probiotic functions and is a promising candidate for a next-generation probiotic^[16]^. Studies have shown that *Faecalibacterium prausnitzii* attenuates CKD through the G protein-coupled receptor 43 signaling^[17]^. Another study revealed that butyrate could suppress the activation of the Janus kinase-signal transducer and activator of transcription (JAK-STAT) pathway by enhancing histone acetylation and promoting the expression of suppressor of cytokine signaling 1^[18]^. *A. muciniphila* can also increase the proportion of *Lactobacillus*, which contains several probiotic strains such as *L. bulgaricus*, *L. plantarum*, *L. rhamnosus*, and *L. casei*, all of which have a long history of use as probiotics^[19]^. These bacteria and their metabolites are essential in nutrition, anti-inflammation, and immune regulation^[20–21]^. However, we were unable to identify the specific strains of *Lactobacillus* that increased in response to *A. muciniphila* pretreatment. Future studies on *Lactobacillus* and AKI are required. We also found a higher abundance of *Alloprevotella* and *Klebsiella* in the AKI group than in the *A. muciniphila*-treated groups. Fecal samples of CKD patients have been reported to be enriched with *Alloprevotella*^[22]^, indicating its role in the progression of kidney diseases. Similarly, *Klebsiella* in the gut produces enterotoxin tilimycin, leading to the apoptosis of intestinal epithelium cells and the progression of colitis^[23–24]^.

The metabolites of the gut microbiota have attracted significant attention because of their roles as signaling molecules and substrates for metabolic reactions^[25]^. A previous study demonstrated that *L. casei Zhang* elevated short-chain fatty acids (SCFAs) and nicotinamide to alleviate renal injury^[12]^. In the present study, we found that *A. muciniphila* altered metabolic pathways while alleviating AKI. These pathways included those associated with tyrosine metabolism; alanine, aspartate, and glutamate metabolism; central carbon metabolism in cancer; GABAergic synapse; pentose and glucuronate interconversions, valine, leucine and isoleucine biosynthesis; prolactin signaling; phenylalanine metabolism; and galactose metabolism. Studies have shown that tyrosine metabolism is impaired in renal disease, necessitating the supplementation of exogenous tyrosine to improve nitrogen balance in patients^[26]^. Pyruvic acid, a metabolite included in the tyrosine metabolism pathway, attenuates renal injury, and its depletion is observed in the renal cortex during AKI^[27]^. The mitochondrial pyruvate carrier (MPC) transports cytosolic pyruvate into mitochondria, promoting the tricarboxylic acid cycle. Previous research revealed that MPC2 overexpression attenuated cisplatin-mediated nephrotoxicity both *in vitro* and *in vivo* by restoring pyruvate metabolism and mitochondrial function^[28]^. In our study, pyruvic acid exhibited higher abundance in the Akk d17 group. It has been demonstrated that pyruvic acid is involved in several altered metabolic pathways, including pentose and glucuronate interconversions, alanine, aspartate, and glutamate metabolism, central carbon metabolism in cancer, valine, leucine and isoleucine biosynthesis, citrate cycle (also known as TCA cycle), and phenylalanine metabolism^[29]^. Additionally, studies reported that succinate, another metabolite involved in metabolic pathways such as alanine, aspartate and glutamate metabolism, central carbon metabolism in cancer, valine, leucine and isoleucine biosynthesis, TCA cycle, and phenylalanine metabolism, had elevated levels and inhibited influenza virus infection and alleviated cardiac reperfusion injury^[30]^. However, another study showed that succinate induced apparent renal injury after 12 weeks of treatment, providing the first evidence that succinate acts as a risk factor contributing to renal injury^[31]^. There are many metabolites, such as pyruvic acid, hydroxylamine, and sulfuric acid, that showed different abundances between the AKI group and the Akk d17 group. Yet, we know little about their roles in AKI. Hence, studies on these specific metabolites are urgently needed, and future research is required to clarify the pathological effects of succinate and other metabolites on renal functions.

The intestinal barrier is closely related to many diseases. It consists of a mucus layer, epithelial cells, and the junctions between them, and the immune barrier. The collapse of the intestinal barrier integrity is marked by mucus degradation, damaged cell contacts, and transformation of cell polarization, which leads to enteric infection, autoimmune disease, and other chronic diseases^[32]^. However, the link between the intestinal barrier and AKI is less well-characterized. Some bacteria and their metabolites can regulate the gut barrier function^[33]^. Microbial metabolites such as SCFAs, bacteriocins, microbial amino acids, conjugated fatty acids, and indole derivatives may also affect gut barrier functions. *A. muciniphila* activates the NF-κB pathway, increasing the expression of *MUC2*, *BIRC3*, and *TNFAIP3* genes, which are involved in gut barrier function^[34]^. The AKI group in this study showed lower expression of MUC2 in the colon tissue, which was restored by *A. muciniphila* treatment. These findings demonstrated that *A. muciniphila* protected the gut barrier function and prevented the translocation of bacteria into circulation, contributing to its protective effects on AKI.

This study has some limitations. The causal correlation between AKI and the gut microbiota is not yet well confirmed and awaits verification of specific strains or metabolites that might be involved in alleviating AKI. Future studies are needed to clarify the role of the intestinal barrier during the onset and progression of AKI, to assess its potential as a therapeutic target, and to validate the effects of metabolites identified through metabolome sequencing (*e.g.*, SCFAs, pyruvic acid, and succinic acid) on AKI progression. Another limitation of this study is the relatively small sample size, which may reduce statistical power and generalizability. Further studies with larger sample sizes are needed to validate and extend these findings.

In summary, this study revealed that *A. muciniphila* alleviated LPS-induced AKI in mice and protected the gut barrier. It also altered the gut microbiota and the associated metabolites. Our findings provide a foundation for further research into the relationship between AKI and the gut microbiota, as well as for targeting the gut microbiota to develop new therapeutic strategies.

## Additional information

The online version contains supplementary materials available at http://www.jbr-pub.org.cn/article/doi/10.7555/JBR.39.20250162.
