# Supplementary material for: Probiotic Akkermansia muciniphila alleviates acute kidney injury by protecting the intestinal barrier and modulating gut microbiota and metabolites
Source: J Biomed Res. 2025 May 28;40(1):76–88. doi: 10.7555/JBR.39.20250162 (PMC12794173; doi:10.7555/JBR.39.20250162)
Supplement: Supplementary file 1 — The online version contains supplementary materials available at http://www.jbr-pub.org.cn/article/doi/10.7555/JBR.39.20250162. [file jbr-40-1-76-S1.pdf]

Available online at www.jbr-pub.org.cn

Open Access at PubMed Central

Journal of Biomedical Research, 2026 40(1): 1–4

Supplementary Data

JBR

Probiotic *Akkermansia muciniphila* alleviates acute kidney injury by protecting the intestinal barrier and modulating gut microbiota and metabolites

Juan Ni[1,2], Zhan Yang[2], Xuewei Sun[2], Qian Cui[3], Ruonan Zhang[4], Han Lu[5], Zihan Wu[2], Jingfeng Zhu[1], Huijuan Mao[1], Kang Liu[1], Chengliang Tang[2], Chunhui Wang[2], Changying Xing[1,✉], Jin Zhu[2,4,✉]

[1]*Department of Nephrology, the First Affiliated Hospital of Nanjing Medical University, Nanjing, Jiangsu 210029, China;*
[2]*Department of Infectious Disease Prevention and Control, Huadong Medical Institute of Biotechniques, Nanjing, Jiangsu 210002, China;*
[3]*Air Force Hospital of Eastern Theater, Nanjing, Jiangsu 210002, China;*
[4]*School of Basic Medical Sciences, Nanjing Medical University, Nanjing, Jiangsu 211166, China;*
[5]*Department of General Surgery, The Second Affiliated Hospital of Nanjing Medical University, Nanjing, Jiangsu 210011, China.*

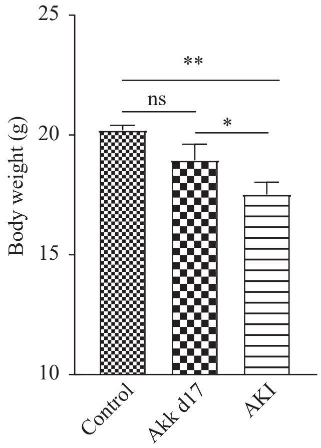

Supplementary Fig. 1 **Body weight of mice on day 17 in each group.** Data are presented as mean ± standard deviation (n = 7 mice per group). $^{*}P < 0.05$ and $^{**}P < 0.01$ by Student's t-test. Abbreviation: ns, not significant.

✉Corresponding authors: Jin Zhu, Department of Infectious Disease Prevention and Control, Huadong Medical Institute of Biotechniques, 293 Zhongshan Road, Nanjing, Jiangsu 210002, China. E-mail: zhujin1968@njmu.edu.cn; Changying Xing, Department of Nephrology, the First Affiliated Hospital of Nanjing Medical University, 300 Guangzhou Road, Nanjing, Jiangsu 210029, China. E-mail: cyxing62@126.com.

Received: 17 April 2025; Revised: 15 May 2025; Accepted: 20 May 2025; Published online: 28 May 2025

CLC number: R692, Document code: A

The authors reported no conflict of interests.

This is an open access article under the Creative Commons Attribution (CC BY 4.0) license, which permits others to distribute, remix, adapt and build upon this work, for commercial use, provided the original work is properly cited.

© 2026 by Journal of Biomedical Research.

https://doi.org/10.7555/JBR.39.20250162

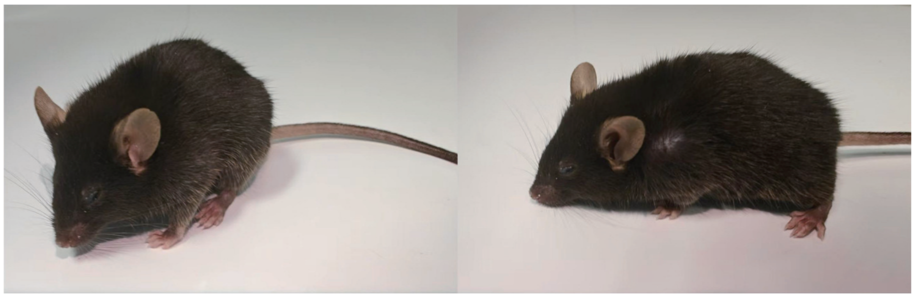

Supplementary Fig. 2 **Representative images of mice in control (left) and AKI (right) groups.** The health and growth status of the male C57BL/6 mice were assessed daily based on activity- and appearance-related parameters. Mice in the AKI group, shown in the right photo, had a poor appetite, slow gait, hair loss, and cloudy conjunctiva.

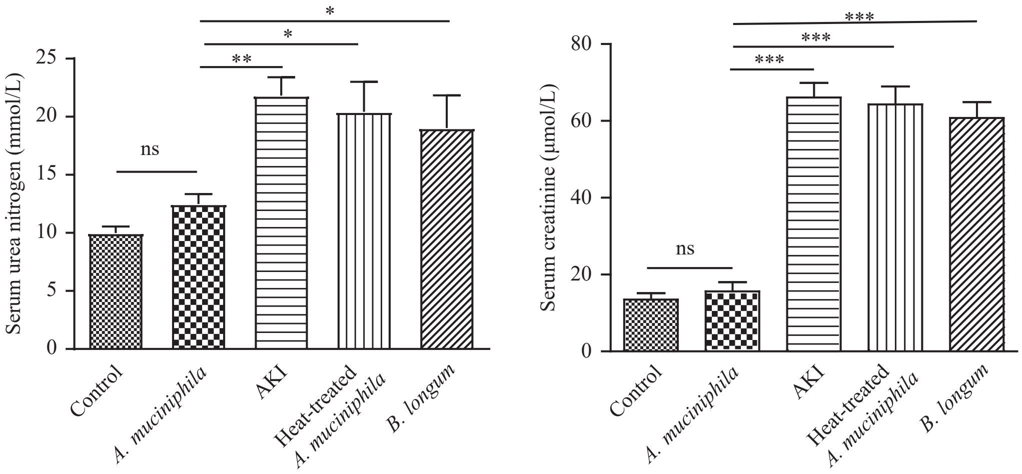

Supplementary Fig. 3 **Serum concentrations of urea nitrogen (left) and creatinine (right) in different groups of mice.** C57BL/6 mice received daily oral gavage of *A. muciniphila*, heat-treated *A. muciniphila*, and *B. longum* (1×10^9 CFU) for 14 days, while mice in the control and AKI groups received daily gavage of sterile PBS (0.2 mL). On day 15, mice in the AKI, the *A.muciniphila*, heat-treated *A. muciniphila,* and *B. longum* groups received a single intraperitoneal injection of LPS (5 mg/kg body weight). Mice in the *A. muciniphila*, the heat-treated *A. muciniphila,* and the *B. longum* groups continued to receive probiotics for 2 days, while mice in the control and AKI groups continued to receive sterile PBS gavage on days 16 and 17. Data are presented as mean ± standard deviation. n = 7 mice per group. $^{*}P < 0.05$, $^{**}P < 0.01$, and $^{***}P < 0.001$ by Student's t-test. Abbreviations: AKI, acute kidney injury; CFU, colony-forming units; LPS, lipopolysaccharide; ns, not significant.

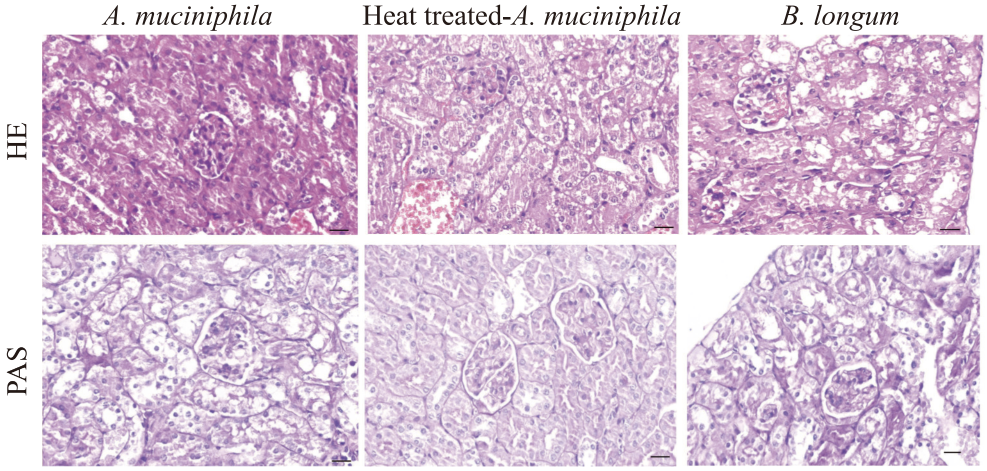

Supplementary Fig. 4 **Representative pathological staining images (HE and PAS) of mouse kidney sections from the *A.muciniphila*, heat-treated *A. muciniphila*, and *B. longum* groups.** Scale bar, 25 μm. Abbreviations: AKI, acute kidney injury; HE, hematoxylin and eosin; PAS, periodic acid–Schiff.

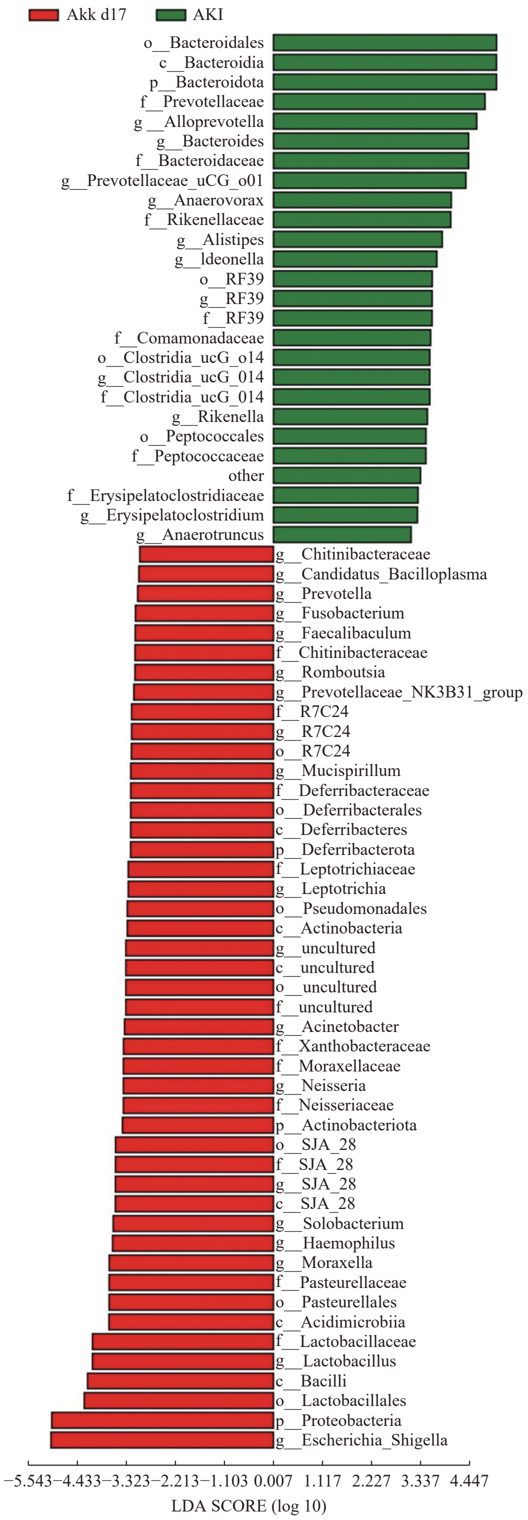

Supplementary Fig. 5 **Fecal samples were collected for 16S rRNA gene sequencing.** Genomic DNA was extracted from colonic contents. Alpha diversity was assessed based on Shannon and Chao1 indices, while beta diversity was visualized *via* principal coordinate analysis and linear discriminant analysis effect size. *n* = 7 for each group. Abbreviations: AKI, acute kidney injury; Akk, *A. muciniphila.*

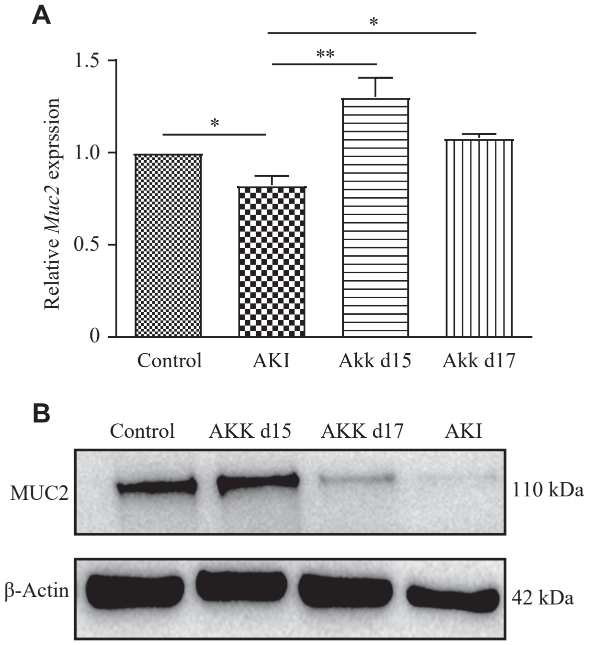

Supplementary Fig. 6 **MUC2 expression in colon tissue among the four groups.** A. Relative *Muc2* mRNA expression quantified by real-time quantitative PCR. B. Protein expression levels of MUC2 quantified by Western blotting. $n = 7$ for each group. $^{*}P < 0.05$, $^{**}P < 0.01$, and $^{***}P < 0.001$ by Student's t-test. Abbreviations: AKI, acute kidney injury; Akk, *A. muciniphila.*

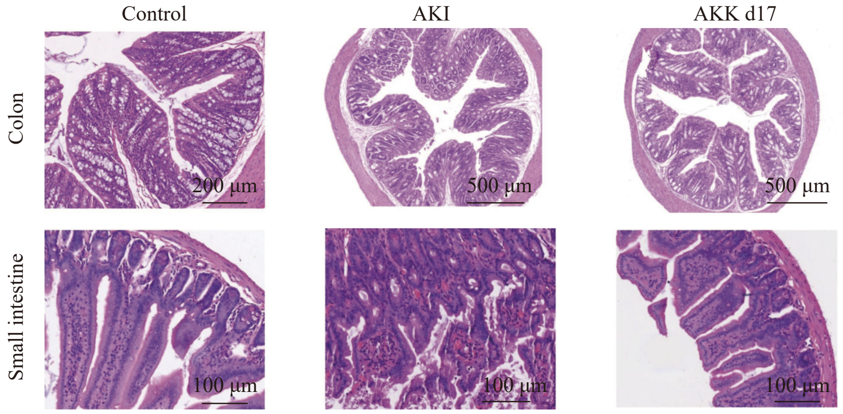

Supplementary Fig. 7 **Representative HE staining images of mouse colon and small intestine tissue sections from the control, AKI, and Akk d17 groups.** Abbreviations: AKI, acute kidney injury; Akk, *A. muciniphila*; HE, hematoxylin and eosin.
